# Effects of Knee Osteoarthritis on Hip and Ankle Gait Mechanics

**DOI:** 10.1155/2019/9757369

**Published:** 2019-03-24

**Authors:** Du Hyun Ro, Joonhee Lee, Jangyun Lee, Jae-Young Park, Hyuk-Soo Han, Myung Chul Lee

**Affiliations:** Department of Orthopaedic Surgery, Seoul National University College of Medicine, Seoul, Republic of Korea

## Abstract

**Introduction:**

Knee osteoarthritis (OA) can affect the hip and ankle joints, as these three joints operate as a kinetic/kinematic chain while walking.

**Purpose:**

This study was performed to compare (1) hip and ankle joint gait mechanics between knee OA and control groups and (2) to investigate the effects of knee gait mechanics on the ipsilateral hip and ankle joint.

**Methods:**

The study group included 89 patients with end-stage knee OA and 42 age- and sex-matched controls without knee pain or OA. Kinetic and kinematic parameters were evaluated using a commercial optoelectric gait analysis system. Range of motion (ROM) during gait, coronal motion arc, and peak joint moment of hip, knee, and ankle joints were investigated.

**Results:**

Ankle varus moment was 50% higher in the OA group (p=0.005) and was associated with higher knee adduction moment (p<0.001). The ROM of the hip and ankle joints were significantly smaller in the OA group and were associated with limited ROM of the knee joint (both p<0.001). The coronal motion arc of the hip was smaller in the OA group and was also associated with limited motion arc of the knee (p<0.001).

**Conclusions:**

Knee OA has a negative effect on the ROM, coronal motion arc, and joint moment of the ankle joint and hip joint. As knee OA is associated with increased moment of the ankle joint, attention should be paid to the ankle joint when treating patients with knee OA.

## 1. Introduction

Knee osteoarthritis (OA) is a leading cause of disability in the elderly population [1]. More than 250 million people suffer from knee OA worldwide, and 1%–2% of the gross national product is spent on OA [2, 3]. Knee OA causes pain and gait disturbances and has a characteristic gait pattern [4, 5].

Knee OA patients have reduced range of motion (ROM) and increased ground reaction force [4, 5]. Stride length and walking speed are decreased in OA [6]. Patients attempt to reduce pain by minimizing the impact on their knees [4, 5]. The gait of knee OA patients is also characterized by higher knee adduction moment (KAM), a marker of medial joint loading and known risk factor for progression of arthritis [7–9]. Such gait changes in knee OA can directly or indirectly affect adjacent weight-bearing joints, i.e., the hip and ankle joints [10–12].

In fact, we often encounter knee OA patients with ankle and hip joint pathology. The joint line orientation angle of the ankle changes and hip arthritis is often observed on X-ray examination [13]. Epidemiological studies also showed that significant numbers of patients have pathologies in more than two of the three weight-bearing joints, which may indicate that problems with one joint are biomechanically related to problems in the others [14, 15]. Many studies have focused on the gait mechanics of the knee in patients with knee arthritis. However, few studies have examined secondary gait changes in adjacent joints, i.e., the hip and ankle joints [10–12]. Theoretically, gait changes in the knee joint can cause gait changes in the hip and ankle joints, as these three joints operate as a kinetic/kinematic chain while walking. Investigation of secondary biomechanical changes in the ankle and hip joints in knee OA patients will provide a better understanding of the gait mechanics of knee OA patients.

In this study, we hypothesized that the gait mechanics of the knee joint would differ between the knee OA group and controls without knee OA, which would have different effects on the hip and ankle joints. This study was performed to compare (1) hip and ankle joint gait mechanics between knee OA patients and controls and (2) to investigate the effects of knee gait mechanics on the ipsilateral hip and ankle joints.

## 2. Methods

### 2.1. Study Subjects

This study was approved by our Institutional Review Board. We retrospectively reviewed 143 patients with three-compartment end-stage OA. End-stage OA was defined as Kellgren–Lawrence (KL) grade 4 in anteroposterior (AP) and lateral knee radiography. We excluded 31 subjects according to the following criteria: (1) aged > 75 or < 55 years (*n *= 26); (2) spine disease, hip or ankle arthritis (KL grade > 2) on X-ray (*n *= 15); (3) male gender (n = 5); (4) inflammatory or traumatic arthritis of the knee (*n *= 4); or (5) any prior bone surgery in the lower extremities (*n *= 4). Consequently, 89 patients with end-stage knee OA were included in the final analysis. The study population had a mean age of 65.8 years, mean height of 151.7 cm, and mean weight of 61.0 kg (Table 1).

The control group consisted of a total of 42 asymptomatic sex- and age-matched volunteers. Gait analysis and X-ray evaluation were performed after receiving informed consent. None of the volunteers experienced concurrent knee pain, had a diagnosis of knee OA, or violated any other exclusion criterion. The controls had a mean age of 64.5 years, mean height of 153.7 cm, and mean weight of 58.2 kg (Table 1).

### 2.2. Gait Analysis Protocol

Gait data were collected from the Human Motion Analysis Lab at our institution. Participants were asked to perform an gentle 5-minute walk to warm up. After warming up, reflective markers were placed on the subjects [16], and they were asked to walk at a self-selected speed along a 9-m track.

Motion (kinematic) data were acquired at a sample rate of 120 Hz using 12 charge-coupled device cameras equipped with a three-dimensional optical motion capture system (Motion Analysis, Santa Rosa, CA, USA). Ground reaction force (kinetic) data were acquired at a sampling rate of 1,200 Hz using three AMTI (Advanced Mechanical Technology Inc., Watertown, MA, USA) force plates. The kinetic data were then normalized by height and weight (% body weight × height) [17].

We used Eva Real-Time software (Motion Analysis), Microsoft Excel 2016 (Microsoft Corp., Redmond, WA, USA), and MATLAB R2017a (MathWorks, Natick, MA, USA) for real-time motion capture, postprocessing, and marker data tracking. The average of three representative strides from five or six separate trials was used for the analysis of each session. Data for the right side of participants were used in the analysis.

Spatiotemporal data are shown in Table 1. The kinematic parameters investigated were the range of motion (ROM) of the hip, knee, and ankle joints, and the coronal motion arcs of the knee and hip joints. Coronal motion arc was defined as the difference in angle between the maximum valgus (or adduction) angle and the maximum varus (or abduction) angle during a stance phase. For kinetics, the peak moments of the sagittal and coronal planes were evaluated. The sagittal plane moment included internal knee extension moment, hip extension/flexion moment, and ankle plantar flexion moment, and the coronal plane moment included the KAM, hip abduction moment, and ankle varus moment.

### 2.3. Radiological Measurement

Radiographic evaluations were performed independently by two authors (both trained in arthroplasty) blinded to other patient information. The interobserver reliability of the radiological assessments was satisfactory (intraclass correlation coefficient, 0.87–0.93). The average values measured by the two observers were used in the analysis. Mechanical axis (hip-knee-ankle axis) was measured using standing full-limb radiography. All radiographic images were digitally acquired using a picture archiving and communication system (PACS) (Maroview 5.4; Infinitt, Seoul, Republic of Korea), and assessments were performed using the PACS software.

### 2.4. Statistical Analysis

Kinetic and kinematic variables were compared using independent Student's* t* test. The normality of the data was assessed using the Kolmogorov–Smirnov test. Correlations were assessed using Pearson's correlation coefficient. The partial correlation coefficient was calculated to control the effect of gait speed on ROM. For all analyses,* p*<0.05 was taken to indicate statistical significance. Statistical analyses were performed using SPSS® for Windows (ver. 19.0.1; SPSS Inc., Chicago, IL, USA).

## 3. Results

**Figure 1:**
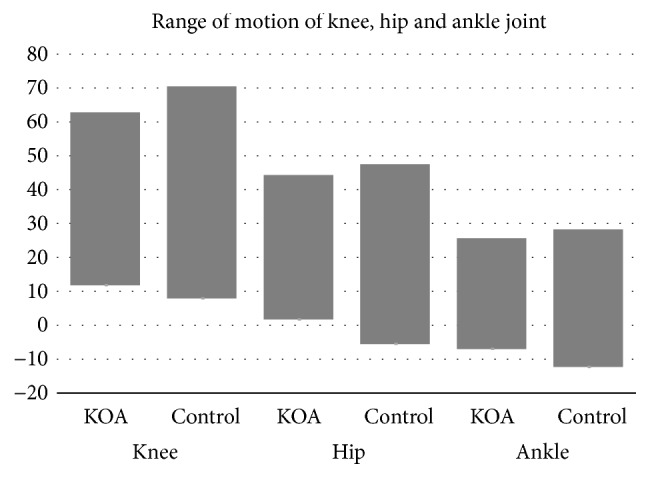
*Range of motion (ROM) of the knee, hip, and ankle joint.* The knee OA (KOA) group showed smaller ROM than the control group. In the knee joint, a negative value indicates hyperextension. In the hip joint, a negative value indicates extension. In the ankle joint, a negative value indicates plantar flexion.

**Figure 2:**
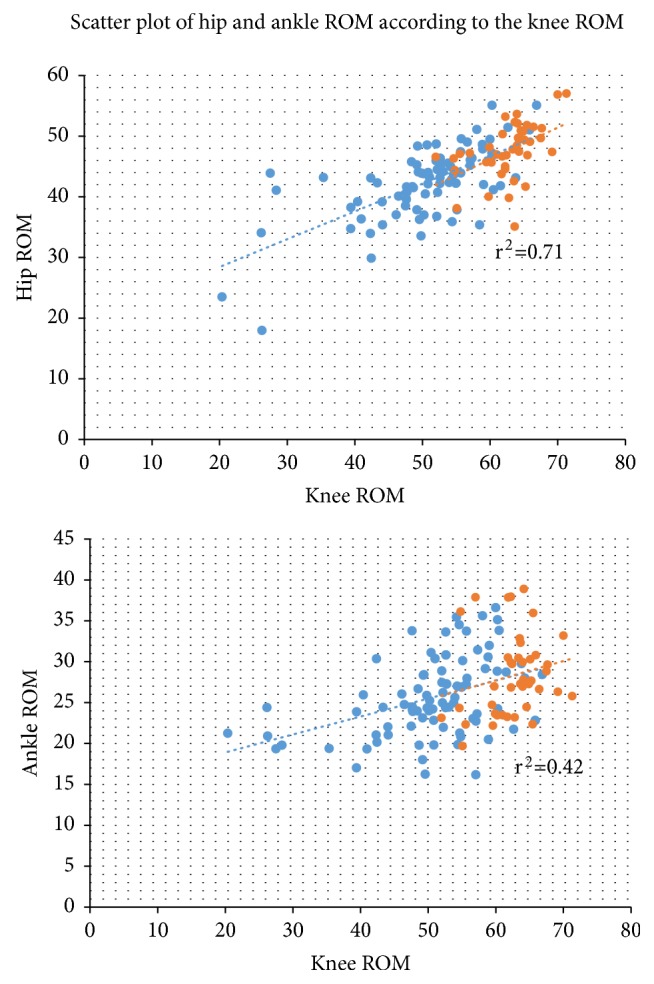
*Correlation between knee ROM and hip and ankle ROM.* Strong correlations were observed between knee ROM and the hip and ankle ROM. Blue dots indicate the knee OA group. Orange dots indicate the control group. Dotted lines indicate the trend lines. r^2^: Pearson correlation coefficient.

The hip and ankle ROM were significantly smaller in the OA group than the control group (Table 2, Figure 1). Knee ROM was also smaller in the OA group (*p*<0.001), and it was correlated with hip and ankle ROM (both* p*<0.001, Figure 2). Controlling the effect on gait speed did not change the fact that knee ROM affected hip and ankle ROM (both* p*<0.001); smaller knee ROM was associated with smaller hip ROM and angle (r^2^ = 0.71–0.42).

**Figure 3:**
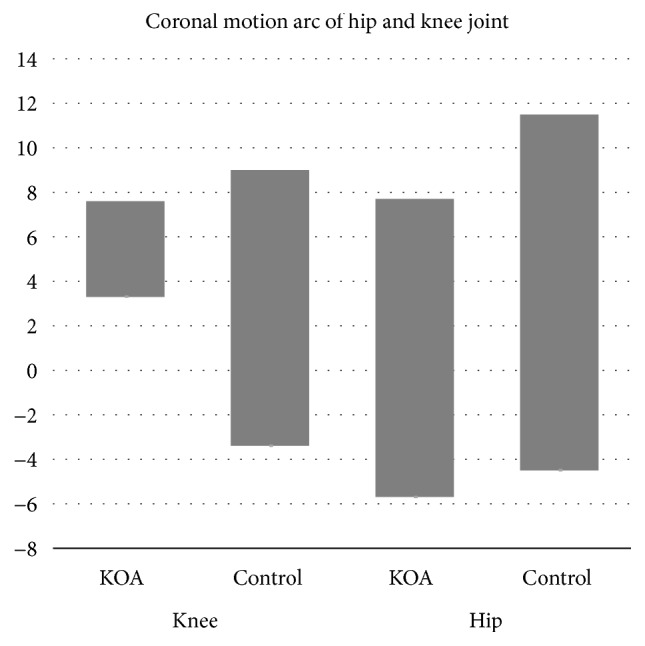
*Coronal motion arcs of the hip and knee joints.* The KOA group showed smaller coronal motion arcs compared to the control group. Negative values indicate valgus and abduction in the knee and hip joints, respectively. There was a significant correlation between the hip and knee joints.

The coronal motion arcs of the hip and knee were also smaller in the OA group than the control group (all* p*<0.001, Figure 3). The coronal motion arc of the knee joint was also correlated with that of the hip joint (r^2^ = 0.36,* p*<0.001).

The sagittal moments of the hip and ankle were smaller in the OA group compared to the controls (Table 3). Sagittal knee moment was also smaller in the OA group than the control group (*p*<0.001).

The coronal moment of the hip was smaller, but the coronal moment of the ankle joint was higher, in the OA group compared to the control group (both* p*=0.005). The ankle varus moment was 50% higher in the OA group than the control group. KAM was also higher in the OA group than the controls (*p*<0.001). The coronal knee moment (KAM) was correlated with those of the hip (r^2^ = 0.19,* p*=0.032) and ankle joints (r^2^ = 0.32,* p*<0.001). Total sagittal moment was smaller in the knee OA group than the control group (*p*<0.001). However, total coronal moment was similar between the two groups (*p*=0.239).

## 4. Discussion

The results of this study showed that changes in gait mechanics in the knee joint have a strong effect on the ROM, coronal motion arc, and joint moment of the ankle and hip joints. ROM and sagittal moment were significantly limited and gait became stiffer in patients with OA. Interestingly, ankle varus moment was 50% higher and associated with an increase of KAM. Such changes are important as they can be risk factors for the subsequent development of secondary arthritis and result in increased pain [10, 13, 18]. Limited ROM of the lower extremities can cause joint stiffness and may result in significant disability [19]. Clinicians should keep these points in mind when planning treatments or in educating knee OA patients. In our knee OA patients, the ROM of the knee was reduced in both the sagittal and coronal planes and was associated with limited ROM of the hip and ankle joints. The mean knee ROM in the OA group was 51°, which was 19% smaller than the knee ROM of 63° in the control group. This reduced the ROM of the hip and ankle joints by 10.4% and 7.1%, respectively. In the clinic, knee OA patients are often seen to walk with bending and stiffness of the hip, knee, and ankle joints. In particular, the extension angle of the hip joint was about 5° in the control group, but there was 2° of flexion contracture in knee OA patients, and these patients have a bent posture. This pattern was reported previously in knee OA patients [19]. Steultjens et al. measured the ROM using a goniometer and reported close relations between the ROM of the hip and knee joint [19]. Our observations were consistent with their results, and we showed that the relationship still exists during gait. As gait is coordinated by work of the muscle and joint, flexion contracture can cause constant contraction of the quadriceps femoris [20]. Thus, knee OA patients will suffer from pain as well as increased quadriceps load during gait [20]. The gait speed can also be reduced by limited ROM of each joint, similar to that in patients with total knee arthroplasty [6]. However, after controlling for the effect of gait speed, limited knee ROM significantly affected hip and ankle ROM.

The limited motion in the coronal plane was also of interest. In this study, the coronal motion arc of the knee in the OA group was 55.6% less than that in the control group. The coronal motion arc was also reduced in the hip joint, which can result in an unstable walking pattern. With limited coronal motion, the patient must balance their body by moving the trunk toward the standing limb. This resembles the Trendelenburg gait and is also a characteristic gait pattern of patients with knee OA [21].

The total sagittal plane moment was reduced in the knee OA group. As the sagittal moment is related to gait speed, it may be related to the slow gait speed of knee OA patients [6]. In contrast, the coronal moment was higher in the knee and ankle joints. Increased KAM has been described in knee OA patients in the literature; it is associated with increased medial joint loading and is a surrogate marker for future progression of arthritis [18]. However, it is interesting that ankle varus moment was increased and associated with KAM. Ankle varus moment was 50% higher in the knee OA group compared to the control group. This can be a risk factor for future progression of arthritis [10, 13, 18]. In fact, we often encounter ankle subluxation in patients with knee OA, and further studies are needed regarding this issue.

This study had some limitations. First, no definitive conclusions on causality can be drawn because of the cross-sectional nature of the study design. A further prospective longitudinal study is warranted to determine the nature of the bidirectional relationship. However, this problem was minimized in this study by excluding patients with KL grade 3 or higher arthritis of the hip and ankle joints. In fact, the incidence rates of hip and ankle joint arthritis are not as high as that for the knee joint, and there is no evidence that hip and ankle arthritis precede knee OA [15]. Therefore, knee OA was thought to influence the mechanics of the hip and ankle joints. Second, generalizability could be limited only to female patients, where this study excluded male patients as there is a significant difference in gait between males and females [22]. However, as only the KAM showed gender differences and there were no differences in joint range of motion or hip angle joint moment, generalization should also be possible to male patients.

## 5. Conclusions

Knee OA has a negative effect on the ROM, coronal motion arc, and joint moment of the ankle joint and hip joint. As knee OA is associated with increased moment of the ankle joint, attention should be paid to the ankle joint when treating patients with knee OA.

## Figures and Tables

**Table 1:** Characteristics of knee OA and control groups.

	Knee OA group (n=89)	Control group (n=42)	p-value
	Mean (SD)	Mean (SD)	
Age, yr	65.8 (5.5)	64.5 (2.9)	0.224
Height, cm	151.7 (5.4)	153.7 (5.2)	0.304
Weight, kg	61 (8.6)	58.2 (7.2)	0.004
Body mass index, kg/m2	26.5 (3.5)	24.6 (2.9)	0.002
HKA axis (right side)	5.9 (3.8)	1.7 (1.8)	<0.001
Gait speed (cm/s)	88.7 (17.5)	111.2 (8.4)	<0.001
Stride length (cm)	97.9 (13.7)	115.7 (7)	<0.001
Step width (cm)	10.3 (3.2)	8.5 (1.8)	<0.001

SD: standard deviation, HKA axis: hip-knee-ankle mechanical axis

**Table 2:** Range of motion and coronal motion arc of each joint.

		Knee OA group (n=89)	Control group (n=42)	p-value
		Mean	Mean	
Range of motion (°)	Knee	51	63	<0.001
	Hip	43	48	<0.001
	Ankle	26	28	0.004

Coronal motion arc (°)	Knee	4	9	<0.001
	Hip	8	12	<0.001

**Table 3:** Mean peak moment of each joint.

		Knee OA group (n=89)	Control group (n=42)	p-value
		Mean (SD)	Mean (SD)	
Sagittal	Knee extension	2.44 (1.16)	3.38 (1.03)	<0.001
	Hip flexion/extension	6.41 (1.19)	6.94 (0.89)	0.010
	Ankle plantar flexion	7.1 (1.15)	7.64 (0.61)	0.005
	Total sagittal moment	15.94 (2.43)	17.97 (1.59)	<0.001

Coronal	Knee adduction	3.27 (0.92)	2.76 (0.65)	0.002
	Hip abduction	4.93 (0.83)	5.34 (0.54)	0.005
	Ankle varus	0.69 (0.44)	0.46 (0.36)	0.005
	Total coronal moment	8.89 (1.58)	8.56 (1.13)	0.239

The unit of moment is %Bw*∗*Ht. SD: standard deviation

## Data Availability

No external data is applicable to this manuscript.
